# Another one bites the plastics

**DOI:** 10.1002/ece3.9332

**Published:** 2022-09-14

**Authors:** Luca Gallitelli, Agnese Zauli, Massimiliano Scalici

**Affiliations:** ^1^ University Roma Tre Rome Italy; ^2^ Independent Researcher Rome Italy

**Keywords:** cetonid, plastic fragmentation, rotten wood, saproxylic insect, soil microplastic, terrestrial macroplastic

## Abstract

Old‐growth forests host a rich diversity of invertebrate assemblages. Among them, saproxylic insects play a fundamental role in the nutrient cycle and ecosystem functioning. In these environments, coevolution between insect and plants have reached a stable equilibrium over millions of years. These delicate ecosystems are threatened mainly by habitat loss and fragmentation, and to date, they have to face the new “plastic threat.” Plastics are widespread in all biomes and ecosystems accumulating throughout the years due to their low degradation rate. Once accumulated, large pieces of plastics can be degraded into smaller particles, the latter representing a great threat to biodiversity and ecosystem health, producing detrimental effects on biota. Since the effects of plastics on terrestrial systems remain largely unexplored, this study aimed at contributing to increasing the knowledge on the interaction between plastics and terrestrial biota. We put our emphasis on the novel and broad topic of plastic degradation by saproxylic beetle larvae, describing how they fragmented macroplastics into microplastics. To investigate whether saproxylic cetonid larvae could degrade expanded polystyrene, we performed an experiment. Thus, we put larvae collected in the field in an expanded polystyrene box. We observed that larvae dug in the thickness of the box fragmenting macroplastics into microplastics and producing a total of 3441 particles. Then, we removed the larvae from the EPS box and isolated them in glass jars filled with natural substrate. The substrate was checked for EPS microplastics previously ingested and now egested by larvae. Additionally, we pointed out that plastics remained attached to cetonid larvae setae, with a mean number of 30.7 ± 12.5 items. Although preliminary, our results highlighted that microplastics attached to saproxylic cetonid larvae might be transported into habitats and transferred along the food web. In conclusion, plastic pollution might affect vulnerable species and ecosystem services representing a risk also for human health.

## Graphical abstract

Macroplastics are degraded by abiotic factors (e.g., UV rays, wind, and rain) but also by biotic ones (e.g., larvae). Thus, this fragmentation process originates in microplastics that can be ingested by organisms and remain attached to cetonid larvae setae after digging into plastics. Then, microplastics may be transferred along with the food web when larvae are eaten by predators, such as hole‐nesting birds.

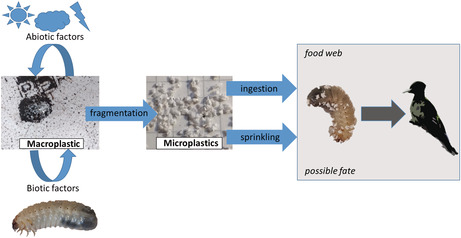

## 1 INTRODUCTION

Old‐growth forests host a rich diversity of invertebrate assemblages. Among them, saproxylic insects play a fundamental role in the nutrient cycle and ecosystem functioning (Stokland et al., 2012). Saproxylic organisms are species that depend on decaying wood in living or dead trees, or other saproxylic organisms during at least one part of their life cycle (Speight, 1989). The evolution of deadwood inhabitants, their specializations, and functional roles (such as woodborers, detrivores, fungivors, predators, and parasites) occurred over millions of years (Stokland et al., 2012). Different assemblages can follow depending on the wood‐decaying stage, setting a complex and well‐established species turnover on this resource. These successional stages imply interactions between organisms that co‐evolved from the Carboniferous when the first saproxylic invertebrate was recorded (Labandeira et al., 1997; Stokland et al., 2012).

In forest ecosystems, saproxylic beetles constitute a large proportion of the biodiversity (Siitonen, 2001) and due to their role as keystone species, they are one of the most important taxa (Sánchez et al., 2017). Indeed, some saproxylic larvae, like the ones of some cetonid beetles, develop in decaying vegetable matter and rotten wood (Micó et al., 2011), thus they are primarily responsible for the mechanical breakdown of woody material, by tunneling and feeding on it (Sánchez et al., 2017), and could be important facilitation factors that determine higher insect species richness in old‐growth forests (Ranius et al., 2005). Plant–animal interaction within old‐growth forests has taken years to establish. These delicate ecosystems and subtle organism interactions were recognized to be threatened mainly due to habitat loss and fragmentation (Grove, 2002). However, at local scale nowadays, they are doomed to face a new threat: plastics. In fact, plastics accumulate in all ecosystems persisting for a long time due to the high durability of polymers (Gallitelli et al., 2020; Rillig, 2012; Strungaru et al., 2019; de Souza Machado et al., 2018). Once accumulated, macroplastics (MA, plastics > 0.5 cm) (Blettler et al., 2018; Gallitelli & Scalici, 2022) can be mechanically fragmented into smaller particles <5 mm, called secondary microplastics (MP) (Thompson et al., 2009).

Based on their size, plastics interact differently with biotic and abiotic ecosystem components and for this reason, MA and MP are studied as two separate units. Nevertheless, as a matter of fact, these are and should be treated as, a continuum in time of the same issue. In fact, ecosystems affected by the occurrence of MA after a certain amount of time, are very likely to be exposed to the impact of autochthonous MP originated from larger debris (Helmberger et al., 2019).

Understanding interactions between MA and biota has a pivotal role in assessing which are the key processes leading to their fragmentation into MP and the potential consequences for the environment. Many studies analyzed how MA can be degraded into MP due to abiotic factors (e.g., wind, UV rays, marine waves, see Andrady, 2011) while only a few focused on the fragmentation caused by biota. Regarding terrestrial ecosystems, the MA fragmentation process is enhanced by fungal and microbial colonization (Kale et al., 2015; Zhang et al., 2019), as well as by insects. For instance, termites and ants were found to be able to degrade polyethylene (PE) MA into MP, and, as they are decomposers of organic matter, can move MP into the soil altering the bioturbation of soil (see Büks et al., 2020). Furthermore, degradation of PE and polystyrene (PS) MA into MP and digestion of MP provoked gut microbiome alteration in Coleoptera Tenebrionidae larvae (Büks et al., 2020; Peng et al., 2019; So et al., 2022; Yang et al., 2015). Oxidative stress, caused by different MP concentrations in the environment, was recorded in Nematoda, Gastropoda, Collembola, and Lumbricidae (see Büks et al., 2020). Other physiological alterations, due to the occurrence of MP in the environment, such as a reduction in motility and body mass, with consequent lower fertility and a decreased life span, were also documented. All these studies suggest that MP affects individuals and with a trickle‐down effect, biological populations and communities, so local diversity and ecosystems (see Büks et al., 2020).

Therefore, as for other ecosystems, the maintenance of healthy old‐growth forests with their well‐settled equilibria is a key for providing services for human welfare. Particularly, old‐growth forests can act as a global carbon sink, indeed they can continue to accumulate carbon (Hoover et al., 2012; Luyssaert et al., 2008) mitigating climate change. Nevertheless, the importance of forest ecosystems is globally recognized, studies on threats to these ecosystems do not take into account the disturbance by plastics, up to now. Thus, this study aimed at increasing the knowledge of interaction between MA and terrestrial biota, pointing out for the first time the possible risk to which old‐growth forests and saproxylic community are exposed to. We carried out an experiment under controlled conditions on macroplastic degradation to investigate whether saproxylic cetonid larvae could degrade expanded polystyrene (hereafter EPS) MA into MP. Here, we put our emphasis on the novel topic of degradation of MA into MP by saproxylic cetonid beetle larvae discussing their role in introducing MP in forest ecosystem food webs.

## 2 MATERIALS AND METHODS

### 2.1 Model organisms

Overall, seven cetonid larvae were collected, in September 2020, by digging in the ground in “Bosco Macchia Grande di Manziana” in central Italy. According to the dichotomous key of Klausnitzer and Krell (1996), larvae of the species sampled were identified as *Protaetia (subgenus Potosia) cuprea* (Fabricius, 1775), tribe Cetoniini (Scarabaeidae, Cetoniinae). Then, to confirm the identification made on larvae, we reared one larva to the adult stage. In forest ecosystems, cetonid larvae are quite common in trunk cavities (Micó et al., 2011). These species are good examples for many other species that show similar ecology and lifestyles indicating good health of forest ecosystems.

### 2.2 First phase of the experiment: Mechanical fragmentation

To investigate whether the collected saproxylic cetonid larvae could degrade EPS MA into MP, we performed an experiment. Larvae were weighed using a digital balance (0.01 g precision) and photographed. Then, larvae were placed in an EPS box with a lid (length 27.0 cm, depth 17.5 cm, height 20.0 cm, thickness 1.50 cm) filled with substrate collected in the field. The substrate is a mixture of rotten wood, decaying vegetable matter, and soil. The rotten wood that constitutes the substrate in the box was of the same size and type as the ones that larvae may colonize in nature. For the EPS box, we bought EPS packaging used for food purposes. The boxes were left outdoor for 7 days and then checked. Outdoor temperatures were measured with a digital thermometer. Temperatures varied between 18.4°C and 28.6°C (mean 23.1°C).

The substrate and larvae were removed from the EPS box. The empty EPS box was checked to find tunnels and holes. When present, EPS fragments were gently removed from the holes with a small brush. To eventually find EPS fragments, the substrate from the EPS box was checked under a stereomicroscope (Nikon C‐LEDS) by zooming up from the minimum magnification (i.e., 0.7×) to the highest one (3.0×).

### 2.3 Second phase of the experiment: Ingestion

After the first phase was completed, each larva was stored in a different 1 L glass jar (10 cm × 10 cm × 10 cm) with a clean substrate. This new substrate was collected in a field with the same composition as the one used in the first phase. Temperature and humidity were kept at an environment level (e.g., 24° and 60%, respectively).

The substrate used in glass jars was checked under a stereomicroscope every 5 days for one month to look for plastic fragments potentially ingested by larvae when still in the EPS box and now egested with feces. Fragments of EPS are easily recognizable for their white color and round shape, and we checked for MP fragments using a stereomicroscope (Nikon C‐LEDS) following the protocol by Gallitelli et al. (2020), Gallitelli et al. (2021). During the experiment we followed protocols to prevent sample contamination, considering EPS particles our target. Specifically, we used nitrile gloves, laboratory coats, and steel tools to avoid external contamination (see Gallitelli et al., 2020).

### 2.4 Data analysis

**FIGURE 1:**
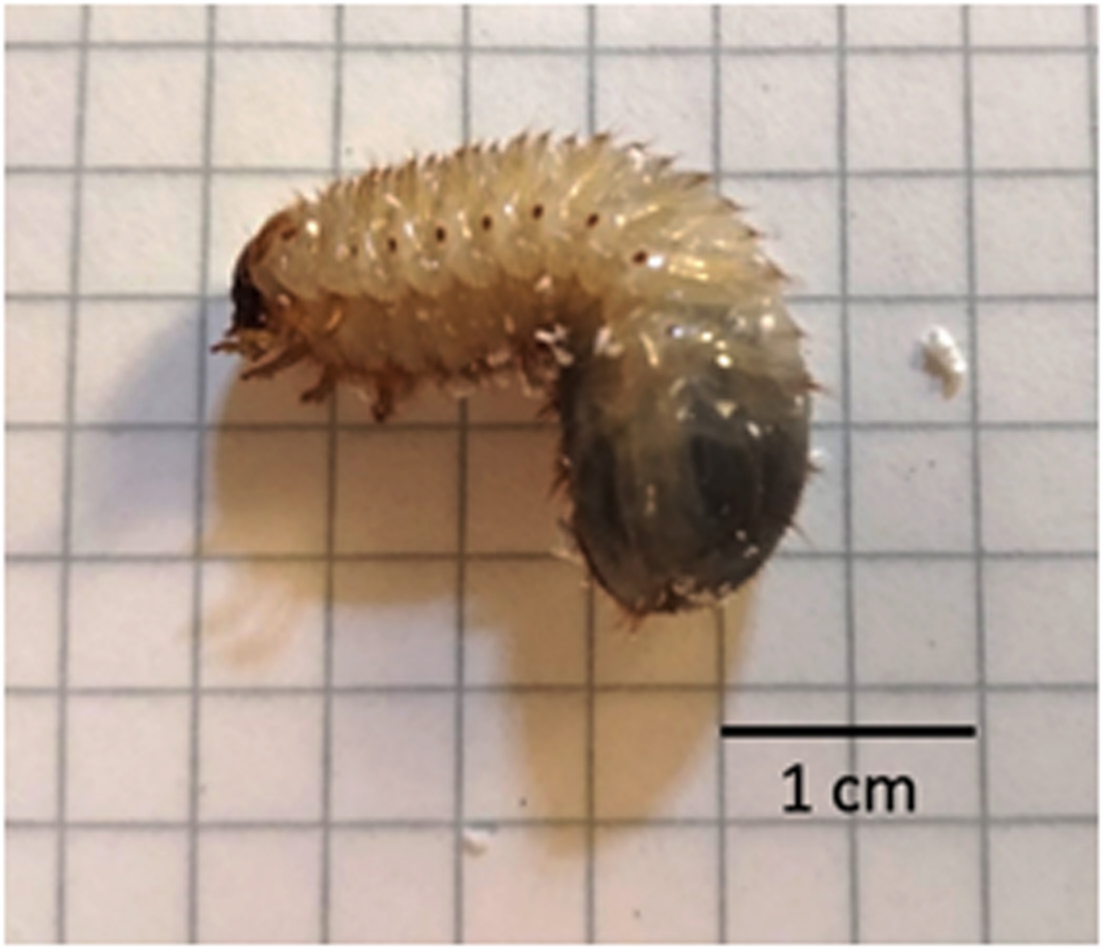
Larvae were photographed to obtain the length of each individual with ImageJ.

Microplastics fragments originated from the EPS box during the first phase of the experiment were weighed and photographed. To count plastic items and measure plastic fragment size, photographs were analyzed with the Image J 1.53c software adapting the protocol by Chen et al., 2021 (Figure S2 in Appendix). In detail, we set the scale for obtaining the size for each particle. To do so, we processed images distinguishing the fluorescence from the background using the default threshold (Chen et al., 2021). As the particles were all approximatively circular, the automated counting function to detect all particles and their features ran easily. All MP features (e.g., number of particles, area, and feret diameter) were reported in Supplementary Materials (Figure S3 and Table S1–S3 in Appendix) (Figure 1).

## 3 RESULTS

In the first phase of the experiment, after 7 days, three larvae were found in the depth of the bottom layer of the EPS box. These larvae dug in the bottom of the EPS box producing three galleries (mean diameter 1.8 cm, overall length 16.5 cm). EPS fragments (*n* = 3441, Figure S1–S3 in Appendix) produced by the digging activity of larvae had irregular rounded shapes with a mean size of 0.27 ± 0.20 cm (feret diameter, see Figure S3 and Table S1–S3 in Appendix). The total volume of fragments was 19.0 ml, and the overall mass was 0.56 g.

**FIGURE 2:**
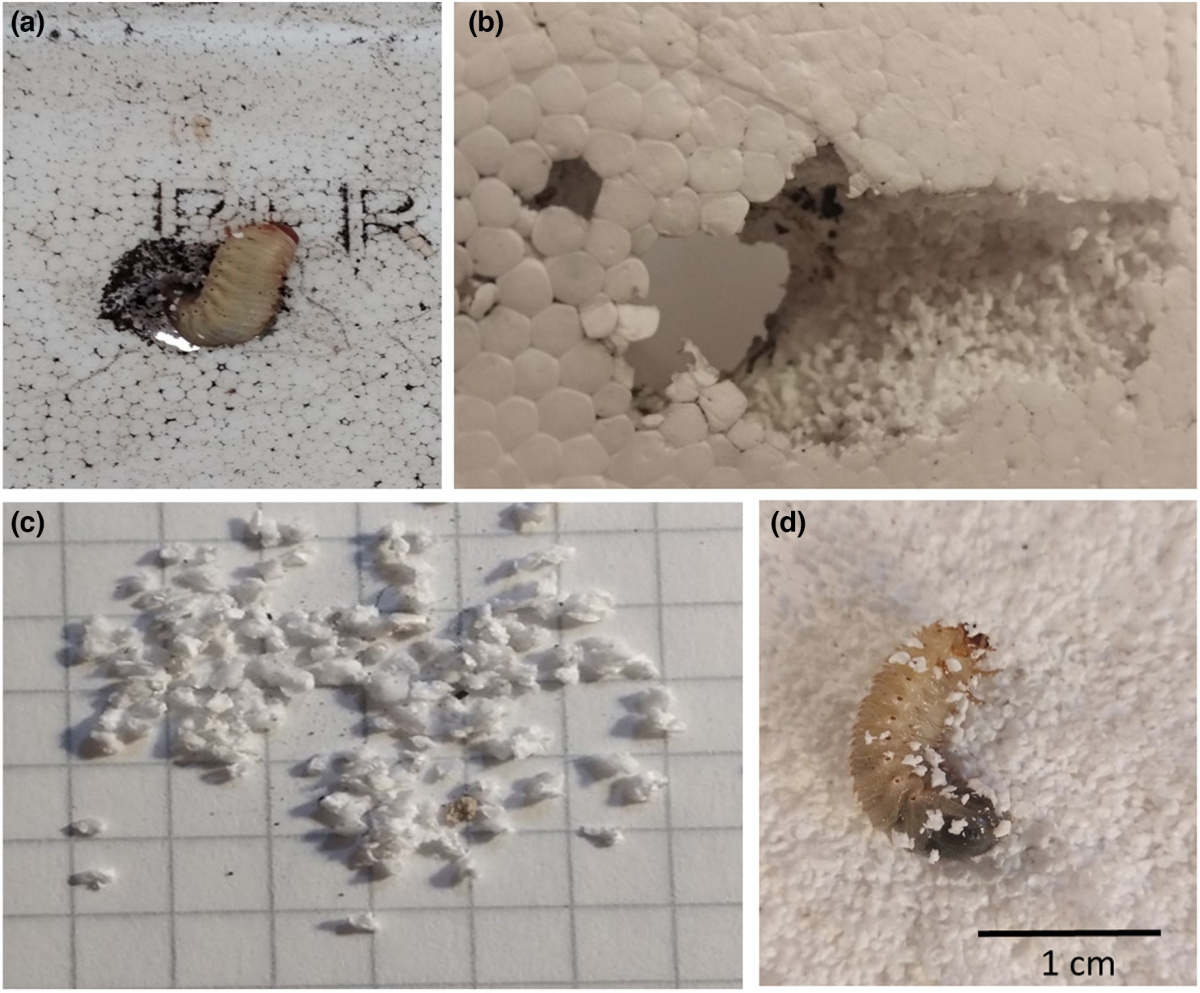
The phases of degrading macroplastics: The entire EPS macroplastic is degraded by larvae (a) originating holes (b) and microplastics (c). EPS MP remained attached to larvae setae (d).

After digging into EPS, larvae remained sprinkled with fragments attached to their setae (Table 1, Figure 2d).

**TABLE 1:** Length and weight of cetonid larvae with the number of MP attached to their body (see Figure 2d)

N° of larva	Length (cm)	Weight (g)	Number of EPS MP
L1	3.28	1.37	39
L2	4.12	1.87	54
L3	3.54	1.61	29
L4	3.86	1.81	31
L5	3.28	1.34	24
L6	3.10	1.39	21
L7	3.19	1.36	17

Concerning the length and the weight of the larvae, the length ranged between 3.10 cm and 4.12 cm, while the weight was between 1.34 g and 1.87 g (Table 1). The mean number of MP attached to their body was 30.7 ± 12.5 items (Figure 2d, Table 1).

No EPS fragments were found in the substrate collected from glass jars used to store larvae in the second phase of the experiment.

## 4 DISCUSSION

This study highlighted for the first time that saproxylic beetle larvae mechanically fragmented EPS MA into MP contributing to filling the knowledge gap on MA degradation in terrestrial ecosystems and on the interaction of MA with terrestrial biota.

Cetonid larvae originated 3441 MP fragments from the EPS box (MA item) within a week. The breaking down of large plastic items is favored by the mouthpart morphology of cetonid larvae apt at cutting and chewing (Video S1). Indeed, although most studies focus on the degradation of MP in terrestrial ecosystems (see Baho et al., 2021; Malizia & Monmany‐Garzia, 2019), cetonid beetles provoked bioerosion of MA, that in natural ecosystems possibly make available autochthonous MP to a wide plethora of terrestrial organisms. Within Coleoptera, it was only found that Tenebrionidae can digest plastic. In particular, *Tenebrio molitor* larvae can biodegrade PS MA within 12–15 h (Büks et al., 2020; Peng et al., 2019; Yang et al., 2015), as well as *Galleria mellonella* (see Lou et al., 2020) and *Zophobas atratus* larvae (Luo et al., 2021).

In addition, fragmentation due to terrestrial biota can allow plastic to enter in a plastic biogeochemical cycle (plastic cycle, see Bank & Hansson, 2019; Brahney et al., 2021). This biotic fragmentation enhances abiotic factors, such as plastic erosion and weathering (e.g., wind, UV rays, and other factors), to degrade plastics more easily. Indeed, it is known that soil microbial communities and terrestrial organisms may accelerate the biodegradation of plastics (Rillig, 2012), and this newly formed MP may affect microbial processes in soils (Rillig et al., 2021).

As known, cetonid larvae can degrade woody substrates facilitating their use by other saproxylic organisms in terrestrial ecosystems (Micó et al., 2011). Therefore, MP made available by cetonids might affect soil fauna and threaten vulnerable species in forest ecosystems as well as the whole saproxylic community that comprehends important indicators of optimal habitat conditions (e.g., listed in the annex II and IV of the Habitats Directive 92/43/EEC). Plastics in terrestrial ecosystems are poorly studied; however, plastics might occur in these habitats (Piehl et al., 2018; Rillig & Lehmann, 2020). Specifically, polystyrene (PS) plastics are broadly used in everyday life activities (Plastics Europe, 2020). Thus, if PS plastics are mismanaged, they could occur in forests and there could be a certain probability of larvae chewing them and fragmenting them into MP. If other polymers (e.g., such as polyethylene terephthalate and polypropylene, respectively) are also occurring within terrestrial ecosystems, larvae may degrade them and PS plastics. Future research is necessary to investigate whether other polymers might be degraded by larvae.

**FIGURE 3:**
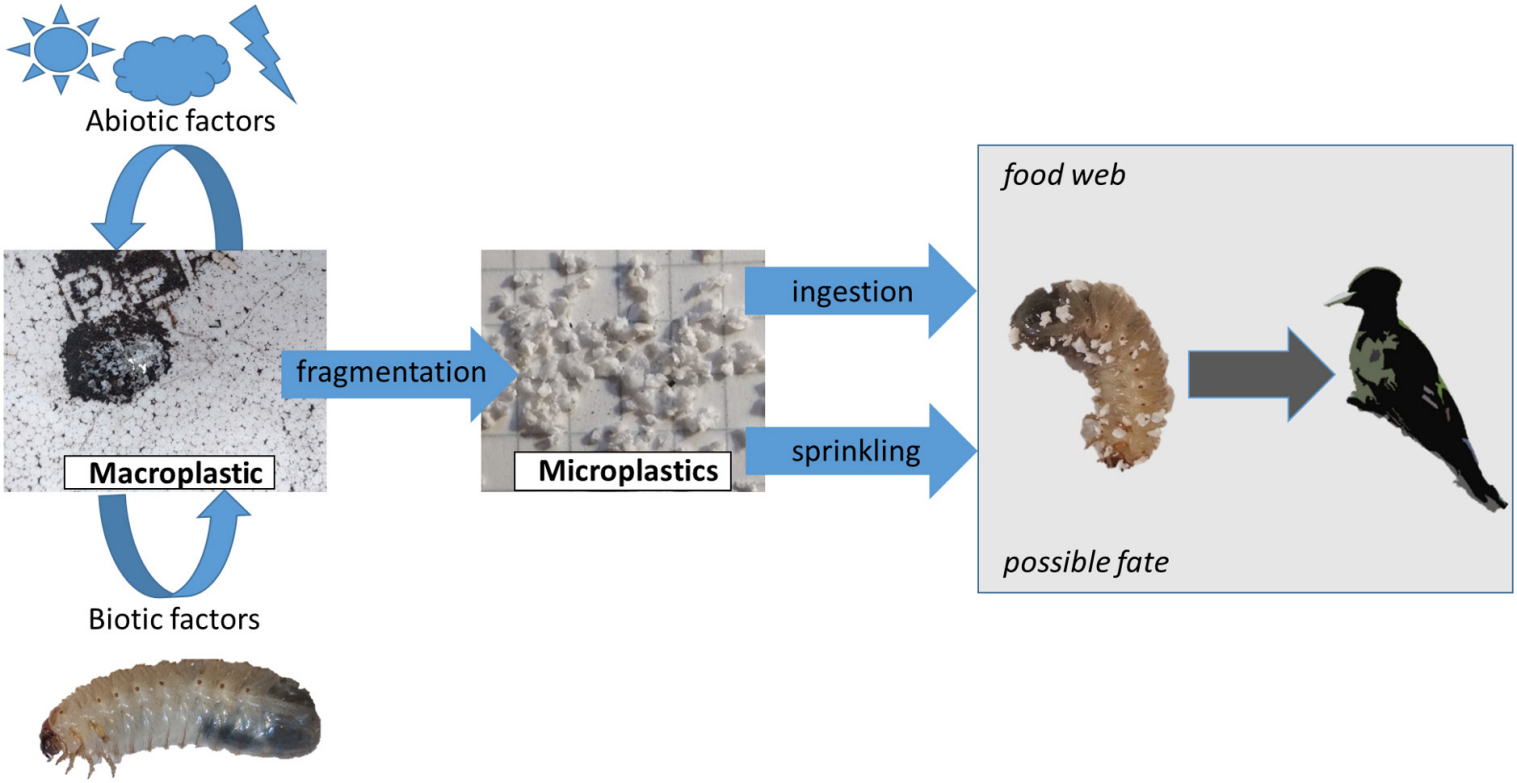
The “plastic cycle”: MA is degraded by abiotic (e.g., UV rays, wind, and rain) and biotic (e.g., larvae) factors. Thus, this fragmentation process originates MP that can be ingested by organisms and be attached to cetonid larvae setae after digging into plastics. Indeed, some terrestrial species were observed to act as bioindicators for soil MP pollution (Al Malki et al., 2021). Then, MP may be transferred along the food chain when the larvae are eaten by predators, such as hole‐nesting birds.

Concerning the second phase of the experiment, as no MP was found in the feces, we assume that the larvae only chewed without ingesting the EPS fragments. Indeed, after digging into EPS, larvae remained sprinkled with fragments attached to their setae. Thus, MP can be transferred along the food chain when the larvae are eaten by predators, such as hole‐nesting birds (Redolfi De Zan et al., 2014) (Figure 3). In addition, due to the long residence time, MP produced by cetonid larvae can enter terrestrial trophic webs (Huerta Lwanga et al., 2017). In fact, saproxylic beetles are at the base of the forest trophic web and they have an important role in recycling energy and matter from deadwood, providing a key ecosystem service.

In conclusion, these preliminary observations provide a new perspective on the bio‐fragmentation of MA in the wider framework of the biogeochemical plastic cycle. Indeed, considering MP, as produced from MA, they can undergo geological processes such as weathering, erosion, and sedimentation provoked by abiotic factors but can also be ingested by organisms, and transferred in the trophic web so affecting ecosystems at different biological levels. Indeed, at the ecosystem level, plastic might impact vulnerable species and ecosystem services being a risk also for human health. However, after presenting our results, it is worth discussing two limitations of our study. First, the sample considered is relatively small, thus future studies should focus on a larger dataset to obtain more robust results. Second, the results we obtained under controlled conditions should be verified in nature. Thus, in this view, future studies should focus on long‐term exposure experiments to investigate the effect of smaller MP on the fitness of saproxylic species and confirm whether insects may be used as an early warning system for MP pollution in forest ecosystems.

## AUTHOR CONTRIBUTIONS

**Agnese Zauli:** Conceptualization (lead); data curation (equal); formal analysis (lead); funding acquisition (supporting); investigation (equal); methodology (equal); supervision (lead); visualization (equal); writing – original draft (equal); writing – review and editing (equal). **Luca Gallitelli:** Conceptualization (lead); data curation (equal); formal analysis (lead); funding acquisition (supporting); investigation (equal); methodology (equal); project administration (equal); software (equal); visualization (supporting); writing – original draft (equal); writing – review and editing (equal). **Massimiliano Scalici:** Conceptualization (equal); funding acquisition (equal); investigation (supporting); project administration (equal); resources (lead); software (equal); supervision (equal); validation (equal); visualization (equal); writing – review and editing (equal).

## FUNDING INFORMATION

This research was supported by the Grant of Excellence Departments, MIUR‐Italy (ARTICOLO 1, COMMI 314–337 LEGGE 232/2016).

## CONFLICT OF INTEREST

The authors have no conflicts of interest to declare.

## Supporting information


Figure S1–S3

Video S1

## Data Availability

Methods and Supplementary files on MP size originated by larvae can be found on Dryad here: https://doi.org/10.5061/dryad.4xgxd2596, https://datadryad.org/stash/share/uDewOosAquxOHSV915_TcBJbQZ_spabwJs1onEwIzu8. *Dryad citation*: Gallitelli, Luca; Zauli, Agnese; Scalici, Massimiliano (2022), Another one bites the plastics, Dryad, Dataset, https://doi.org/10.5061/dryad.4xgxd2596.

## References

[ece39332-bib-0001] Al Malki, J. S. , Hussien, N. A. , Tantawy, E. M. , Khattab, Y. , & Mohammadein, A. (2021). Terrestrial biota as bioindicators for microplastics and potentially toxic elements. Coatings, 11(10), 1152. 10.3390/coatings11101152 PMC8384083 34485701

[ece39332-bib-0002] Andrady, A. L. (2011). Microplastics in the marine environment. Marine Pollution Bulletin, 62(8), 1596–1605. 10.1016/j.marpolbul.2011.05.030 21742351

[ece39332-bib-0003] Baho, D. L. , Bundschuh, M. , & Futter, M. N. (2021). Microplastics in terrestrial ecosystems: Moving beyond the state of the art to minimize the risk of ecological surprise. Global Change Biology, 27(17), 3969–3986. 10.1111/gcb.15724 34042229

[ece39332-bib-0004] Bank, M. S. , & Hansson, S. V. (2019). The plastic cycle: A novel and holistic paradigm for the Anthropocene. Environmental Science & Technology, 53(13), 7177–7179. 10.1021/acs.est.9b02942 31198029

[ece39332-bib-0005] Blettler, M. C. M. , Abrial, E. , Khan, F. R. , Sivri, N. , & Espinola, L. A. (2018). Freshwater plastic pollution: Recognizing research biases and identifying knowledge gaps. Water Research, 143, 416–424. 10.1016/j.watres.2018.06.015 29986250

[ece39332-bib-0006] Brahney, J. , Mahowald, N. , Prank, M. , Cornwell, G. , Klimont, Z. , Matsui, H. , & Prather, K. A. (2021). Constraining the atmospheric limb of the plastic cycle. Proceedings of the National Academy of Sciences of the United States of America, 118(16), e2020719118. 10.1073/pnas.2020719118 33846251 PMC8072239

[ece39332-bib-0007] Büks, F. , Loes van Schaik, N. , & Kaupenjohann, M. (2020). What do we know about how the terrestrial multicellular soil fauna reacts to microplastic? The Soil, 6(2), 245–267. 10.5194/soil-6-245-2020

[ece39332-bib-0008] Chen, S. , Li, Y. , Mawhorter, C. , & Legoski, S. (2021). Quantification of microplastics by count, size and morphology in beverage containers using Nile red and ImageJ. Journal of Water and Health, 19(1), 79–88. 10.2166/wh.2020.171

[ece39332-bib-0009] de Souza Machado, A. A. , Kloas, W. , Zarfl, C. , Hempel, S. , & Rillig, M. C. (2018). Microplastics as an emerging threat to terrestrial ecosystems. Global Change Biology, 24(4), 1405–1416. 10.1111/gcb.14020 29245177 PMC5834940

[ece39332-bib-0010] Gallitelli, L. , Cera, A. , Cesarini, G. , Pietrelli, L. , & Scalici, M. (2021). Preliminary indoor evidences of microplastic effects on freshwater benthic macroinvertebrates. Scientific Reports, 11(1), 1–11. 10.1038/s41598-020-80606-5 33436879 PMC7803787

[ece39332-bib-0011] Gallitelli, L. , Cesarini, G. , Cera, A. , Sighicelli, M. , Lecce, F. , Menegoni, P. , & Scalici, M. (2020). Transport and deposition of microplastics and Mesoplastics along the river course: A case study of a Small River in Central Italy. Hydrology, 7(4), 90. 10.3390/hydrology7040090

[ece39332-bib-0012] Gallitelli, L. , & Scalici, M. (2022). Riverine macroplastic gradient along watercourses: A global overview. Frontiers in Environmental Science, 10, 937944. 10.3389/fenvs.2022.937944

[ece39332-bib-0013] Grove, S. J. (2002). Saproxylic insect ecology and the sustainable management of forests. Annual Review of Ecology, Evolution, and Systematics, 33(1), 1–23. 10.1146/annurev.ecolsys.33.010802.150507

[ece39332-bib-0014] Helmberger, M. S. , Tiemann, L. K. , & Grieshop, M. J. (2019). Towards an ecology of soil microplastics. Functional Ecology, 34(3), 550–560. 10.1111/1365-2435.13495

[ece39332-bib-0015] Hoover, C. M. , Leak, W. B. , & Keel, B. G. (2012). Benchmark carbon stocks from old‐growth forests in northern New England, USA. Forest Ecology and Management, 266, 108–114. 10.1016/j.foreco.2011.11.010

[ece39332-bib-0016] Huerta Lwanga, E. , Mendoza Vega, J. , Ku Quej, V. , Chi J de los, A. , Sanchez del Cid, L. , Chi, C. , Segura, G. E. , Gertsen, H. , Salánki, T. , van der Ploeg, M. , Koelmans, A. A. , & Geissen, V. (2017). Field evidence for transfer of plastic debris along a terrestrial food chain. Scientific Reports, 7(1), 1–7. 10.1038/s41598-017-14588-2 29074893 PMC5658418

[ece39332-bib-0017] Kale, S. K. , Deshmukh, A. G. , Dudhare, M. S. , & Patil, V. B. (2015). Microbial degradation of plastic: A review. Journal of Biochemical Technology, 6(2), 952–961.

[ece39332-bib-0018] Klausnitzer, B. , Krell, F. T. (1996) 6. Überfamilie Scarabaeoidea. Die Käfer Mitteleuropas, Bd. L3: Die Larven der Käfer Mitteleuropas. 3. Band. Polyphaga.

[ece39332-bib-0019] Labandeira, C. C. , Phillips, T. L. , & Norton, R. A. (1997). Oribatid mites and the decomposition of plant tissues in Paleozoic coal‐swamp forests. PALAIOS, 12(4), 319–353. 10.2307/3515334

[ece39332-bib-0020] Lou, Y. , Ekaterina, P. , Yang, S. S. , Lu, B. , Liu, B. , Ren, N. , Corvini, F. , & Xing, D. (2020). Biodegradation of polyethylene and polystyrene by greater wax moth larvae (*Galleria mellonella* L.) and the effect of co‐diet supplementation on the core gut microbiome. Environmental Science & Technology, 54(5), 2821–2831. 10.1021/acs.est.9b07044 32013402

[ece39332-bib-0021] Luo, L. , Wang, Y. , Guo, H. , Yang, Y. , Qi, N. , Zhao, X. , Gao, S. , & Zhou, A. (2021). Biodegradation of foam plastics by *Zophobas atratus* larvae (coleoptera: Tenebrionidae) associated with changes of gut digestive enzymes activities and microbiome. Chemosphere, 282, 131006. 10.1016/j.chemosphere.2021.131006 34118623

[ece39332-bib-0022] Luyssaert, S. , Schulze, E. D. , Börner, A. , Knohl, A. , Hessenmöller, D. , Law, B. E. , Ciais, P. , & Grace, J. (2008). Old‐growth forests as global carbon sinks. Nature, 455(7210), 213–215. 10.1038/nature07276 18784722

[ece39332-bib-0023] Malizia, A. , & Monmany‐Garzia, A. C. (2019). Terrestrial ecologists should stop ignoring plastic pollution in the Anthropocene time. Science of the Total Environment, 668, 1025–1029. 10.1016/j.scitotenv.2019.03.044 31018444

[ece39332-bib-0024] Micó, E. , Juárez, M. , Sánchez, A. , & Galante, E. (2011). Action of the saproxylic scarab larva *Cetonia aurataeformis* (coleoptera: Scarabaeoidea: Cetoniidae) on woody substrates. Journal of Natural History, 45(41–42), 2527–2542. 10.1080/00222933.2011.596953

[ece39332-bib-0025] Peng, B. Y. , Su, Y. , Chen, Z. , Chen, J. , Zhou, X. , Benbow, M. E. , Criddle, C. S. , Wu, W. , & Zhang, Y. (2019). Biodegradation of polystyrene by dark (*Tenebrio obscurus*) and yellow (*Tenebrio molitor*) mealworms (coleoptera: Tenebrionidae). Environmental Science & Technology, 53(9), 5256–5265. 10.1021/acs.est.8b06963 30990998

[ece39332-bib-0026] Piehl, S. , Leibner, A. , Löder, M. G. J. , Dris, R. , Bogner, C. , & Laforsch, C. (2018). Identification and quantification of macro‐ and microplastics on an agricultural farmland. Scientific Reports, 8(1), 1–9. 10.1038/s41598-018-36172-y 30560873 PMC6299006

[ece39332-bib-0027] Plastics Europe (2020). 2020 PlasticsEuropePlastics – the facts 2020 https://www.plasticseurope.org/en/resources/publications/4312‐plastics‐facts‐2020

[ece39332-bib-0028] Ranius, T. , Ekvall, H. , Jonsson, M. , & Bostedt, G. (2005). Cost‐efficiency of measures to increase the amount of coarse woody debris in managed Norway spruce forests. Forest Ecology and Management, 206(1–3), 119–133. 10.1016/j.foreco.2004.10.061

[ece39332-bib-0029] Redolfi De Zan, L. , Battisti, C. , & Carpaneto, G. (2014). Bird and beetle assemblages in relict beech forests of Central Italy: A multi‐taxa approach to assess the importance of dead wood in biodiversity conservation. Community Ecology, 15(2), 235–245. 10.1556/comec.15.2014.2.12

[ece39332-bib-0030] Rillig, M. C. (2012). Microplastic in terrestrial ecosystems and the soil? Environmental Science & Technology, 46(12), 6453–6454. 10.1021/es302011r 22676039

[ece39332-bib-0031] Rillig, M. C. , & Lehmann, A. (2020). Microplastic in terrestrial ecosystems. Science, 368(6498), 1430–1431. 10.1126/science.abb5979 32587009 PMC7115994

[ece39332-bib-0032] Rillig, M. C. , Leifheit, E. , & Lehmann, J. (2021). Microplastic effects on carbon cycling processes in soils. PLoS Biology, 19(3), e3001130. 10.1371/journal.pbio.3001130 33784293 PMC8009438

[ece39332-bib-0033] Sánchez, A. , Micó, E. , Galante, E. , & Juárez, M. (2017). Chemical transformation of Quercus wood by Cetonia larvae (Coleoptera: Cetoniidae): An improvement of carbon and nitrogen available in saproxylic environments. European Journal of Soil Biology, 78, 57–65. 10.1016/j.ejsobi.2016.12.003

[ece39332-bib-0034] Siitonen, J. (2001). Forest management, coarse Woody debris and saproxylic organisms: Fennoscandian boreal forests as an example. Ecological Bulletins, 49, 11–41.

[ece39332-bib-0035] So, M. W. K. , Vorsatz, L. D. , Cannicci, S. , & Not, C. (2022). Fate of plastic in the environment: From macro to nano by macrofauna. Environmental Pollution, 300, 118920. 10.1016/j.envpol.2022.118920 35131331

[ece39332-bib-0036] Speight, M. C. (1989). Saproxylic invertebrates and their conservation. Council of Europe.

[ece39332-bib-0037] Stokland, J. N. , Siitonen, J. , & Jonsson, B. G. (2012). Biodiversity in dead wood. Cambridge University Press.

[ece39332-bib-0038] Strungaru, S. A. , Jijie, R. , Nicoara, M. , Plavan, G. , & Faggio, C. (2019). Micro‐ (nano) plastics in freshwater ecosystems: Abundance, toxicological impact and quantification methodology. Trends in Analytical Chemistry, 110, 116–128. 10.1016/j.trac.2018.10.025

[ece39332-bib-0039] Thompson, R. C. , Moore, C. J. , vom Saal, F. S. , & Swan, S. H. (2009). Plastics, the environment and human health: Current consensus and future trends. Philosophical Transactions of the Royal Society B, 364(1526), 2153–2166. 10.1098/rstb.2009.0053 PMC2873021 19528062

[ece39332-bib-0040] Yang, Y. , Yang, J. , Wu, W.‐M. , Zhao, J. , Song, Y. , Gao, L. , Yang, R. , & Jiang, L. (2015). Biodegradation and mineralization of polystyrene by plastic‐eating mealworms: Part 1. Chemical and physical characterization and isotopic tests. Environmental Science & Technology, 49(20), 12080–12086. 10.1021/acs.est.5b02661 26390034

[ece39332-bib-0041] Zhang, M. , Zhao, Y. , Qin, X. , Jia, W. , Chai, L. , Huang, M. , & Huang, Y. (2019). Microplastics from mulching film is a distinct habitat for bacteria in farmland soil. Science of the Total Environment, 688, 470–478. 10.1016/j.scitotenv.2019.06.108 31254812

